# Combined-modality treatment improved outcome in sinonasal undifferentiated carcinoma: single-institutional experience of 21 patients and review of the literature

**DOI:** 10.1007/s00405-012-2008-5

**Published:** 2012-04-03

**Authors:** Abrahim Al-Mamgani, Peter van Rooij, Robert Mehilal, Lisa Tans, Peter C. Levendag

**Affiliations:** 1 Department of Radiation Oncology, Erasmus MC-Daniel den Hoed Cancer Center, Groene Hilledijk 301, 3075 EA Rotterdam, The Netherlands; 2 Department of Biostatistics, Erasmus MC-Daniel den Hoed Cancer Center, Rotterdam, The Netherlands

**Keywords:** SNUC, IMRT, Chemotherapy, Toxicity, Head and neck cancer

## Abstract

The optimal treatment of sinonasal undifferentiated carcinoma (SNUC) remains unclear. We report our results on the outcome and toxicity of patients with SNUC treated by a combined modality and attempt to define the optimal treatment strategies by reviewing the literature. Between 1996 and 2010, 21 consecutive patients with SNUC were treated by any combination of surgery, chemotherapy and radiotherapy. End points were local control (LC), regional control (RC), disease-free (DFS), cause-specific (CSS) overall survival (OS), and late toxicity. Organ preservation was defined as visual preservation without orbital exenteration. After median follow-up of 54 months, the 5-year actuarial rates of LC, RC, DFS, CSS, and OS were 80, 90, 64, 74, and 74 % respectively. On multivariate analysis, T-stage and multimodality treatment approach correlated significantly with LC. Elective nodal irradiation was given to 42 % of high-risk node-negative patients. None of them developed regional failure. The overall 5-year incidence of grade ≥2 late toxicity was 30 %. Treatment-related blindness was significantly decreased in patients treated with intensity-modulated radiotherapy (IMRT), compared to 2D and 3D-conformal radiotherapy (3DCRT), with organ preservation rates of 86 and 14 % respectively (*p* = 0.006). We concluded that combined-modality treatment with three, or at least two, modalities resulted in good LC, but with high overall rate of late toxicity. However, the incidence of late toxicity and permanent visual impairment were decreased over time by the introduction of IMRT. Because of the improvement in therapeutic ratio achieved by using IMRT, this highly conformal radiation technique should be the standard of care in patients with SNUC.

## Introduction

Sinonasal undifferentiated carcinoma (SNUC) is a rare type of malignancy which was first described by Frierson et al. [1]. SNUCs are usually presented at locally advanced stage involving the nasal cavity and paranasal sinuses with a propensity to extend into the (peri) orbital tissues and the central nervous system. In most series, 10–30 % of patients have a node-positive disease at diagnosis [2–8]. The original reports on SNUC by Frierson et al. [1] painted a grim picture because of the aggressive nature of the disease and the limited knowledge about treatment. Early reports on radiation or surgical resection alone have generally yielded poor results [1, 9]. By multimodality therapy many patients achieve complete remission and prolonged survival, with a significant number potentially cured. Although different combination of surgery, chemotherapy and radiotherapy are nowadays used [2–8], there is still no consensus about which modalities to use and the best sequence.

In the current study we report on a long-term single-institutional experience of the treatment of SNUC by combined modality. We also performed an exact logistic regression analysis to identify the parameters predictive of local failure and reviewed the literature.

## Materials and methods

Between September 1996 and September 2010, 21 consecutive patients with SNUC were treated with curative intent at our institution by any combination of surgery, radiotherapy, and chemotherapy (Table 1). Patients with recurrent or metastatic disease at presentation were excluded from this analysis.

**Table 1:** Patient, tumor, and treatment characteristics (*n* = 21)

	No. of patients (%)
Gender	
Male	11 (52 %)
Female	10 (48 %)
Age (years)	
Range	26–78
Median	52
Follow-up (months)	
Range	4–163
Median	54
Tumor stage^a^	
T3	6 (29 %)
T4a	6 (29 %)
T4b	9 (42 %)
Nodal stage^a^	
N0	19 (90 %)
N+	2 (10 %)
Site	
Ethmoid sinus	16 (76 %)
Maxillary sinus	5 (24 %)
Dural or orbital invasion	
No	14 (67 %)
Dural invasion	3 (13 %)
Orbital invasion	4 (20 %)
Type of treatment	
Primary CRT	7 (33 %)
Induction CT, surgery, and PORT	7 (33 %)
Surgery and PORT	5 (24 %)
Surgery and POCRT	2 (10 %)
Radiation dose (Gy)	
Range	50–70
Median	62.5
Technique radiotherapy	
2D conventional RT	2 (10 %)
3DCRT	5 (24 %)
IMRT	14 (66 %)

*PORT* post-operative radiotherapy; *POCRT* post-operative chemoradiotherapy; *CRT* chemoradiotherapy; *RT* radiotherapy; *3DCRT* three-dimensional conformal RT; *IMRT* intensity-modulated RT

^a^Patients were staged according to the AJCC staging system for nasal cavity and ethmoid sinus (Greene F, Page D, Fleming I, editors. American Joint Committee on Cancer, Nasal Cavity and Paranasal Sinuses (AJCC Cancer Staging Manual), New York, Springer-Verlag; 2002, pp 61–62

Pre-treatment evaluation consisted of complete history and physical examination. All patients had chest X-ray, ultrasonography with fine-needle aspiration, and head and neck MRI or CT scan. All patients were presented in our weekly multidisciplinary head and neck conference. Based on the joint recommendations of that meeting, patients were selected for treatment with surgery followed by (chemo) radiotherapy, induction chemotherapy followed by surgery and radiotherapy, or definitive (chemo) radiotherapy. The goal of surgery is complete resection of the tumor with negative margins, with as low morbidity as possible. The type and extension of the surgery is dictated by the extent of the disease, and cosmetic and functional considerations. The surgical techniques ranged from (sub) total maxillectomy and/or ethmoidectomy to craniofacial resection. Endoscopes were used when reduction of morbidity or improved (angled) visualization could be achieved. Orbital exenteration is not an elective procedure but mandatory in patients with tumor invasion beyond the periorbital tissue. Criteria for unresectability were extensive intracranial or intradural spread, invasion of the optic pathway, invasion of cavernous sinus, and encasement of the carotid artery. These patients are offered either induction chemotherapy followed by surgery and postoperative radiotherapy (PORT) or definitive (chemo) radiation.

### Radiotherapy

Patients were immobilized in a supine treatment position in a custom-made mask. A computer tomography simulation, with a 2.5-mm slice thickness, was performed. During the time span of the study, three different radiation techniques were used (Table 1). Two patients received radiotherapy using a two-dimensional three-field technique (one anterior and two laterals wedged). The lens was shielded at the anterior portal. After 54 Gy, the optic pathway was excluded. However, when the tumor was located very close to or invaded the optic apparatus, a dose of >54 Gy to the optic pathway was given, after discussion with patient. In five patients, 3DCRT was used. From 2002 onward, a highly conformal radiation technique, intensity-modulated radiotherapy (IMRT), was introduced to treat all head and neck cancer patients at our institution. Since that time, 14 patients (66 %) were treated by means of IMRT. The clinical target volume (CTV) to the planning target volume (PTV) margin was 5 mm for 3DCRT and IMRT. Radiotherapy was given at a total dose of 50–70 Gy in the 2-Gy fraction daily in case of 3DCRT and IMRT. According to the local protocol for the treatment of paranasal sinus cancers, elective nodal irradiation (ENI) was advocated in high-risk node-negative patients with involvement of the skin of the cheek, infratemporal fossa, pterygoid, or cribriform plates.

### Chemotherapy

Sixteen patients (76 %) received chemotherapy. Concurrent chemoradiotherapy (*n* = 9) (seven definitive and two postoperative) was given using two cycles of cisplatin (100 mg/m^2^, day 1 and 21) and induction chemotherapy (*n* = 7) using four cycles of cisplatin (80 mg/m^2^ day 1) and etoposide (100 mg/m^2^ day 1, 2 and 3).

### End points

End points of the study were rates of local control (LC), regional control (RC), and disease-free (DFS), cause-specific (CSS) and overall survival (OS). The late toxicity scores were retrospectively collected from chart review using common terminology criteria for adverse events v3.0 (CTCAE). Organ preservation was defined as visual preservation without orbital exenteration.

### Follow-up

After completion of treatment, patients were followed up 2-monthly for the first year, 3-monthly for the second and third year, and 6-monthly thereafter. At each visit, history and clinical examinations were performed.

### Statistical analysis

Survival rates were calculated from the completion of treatment using Kaplan–Meier technique. Possible predictive clinicopathological factors for local failure were tested using exact logistic regression model. All significant tests were two sided and *p* values <0.05 were considered to be statistically significant.

## Results

The patient’s characteristics are shown in Table 1. The tumor was occupying at least two paranasal sinuses. However, the tumor epicenter was in the ethmoid sinus in 76 %, and the maxillary sinus in 24 % of patients.

### Outcomes

**Fig. 1:**
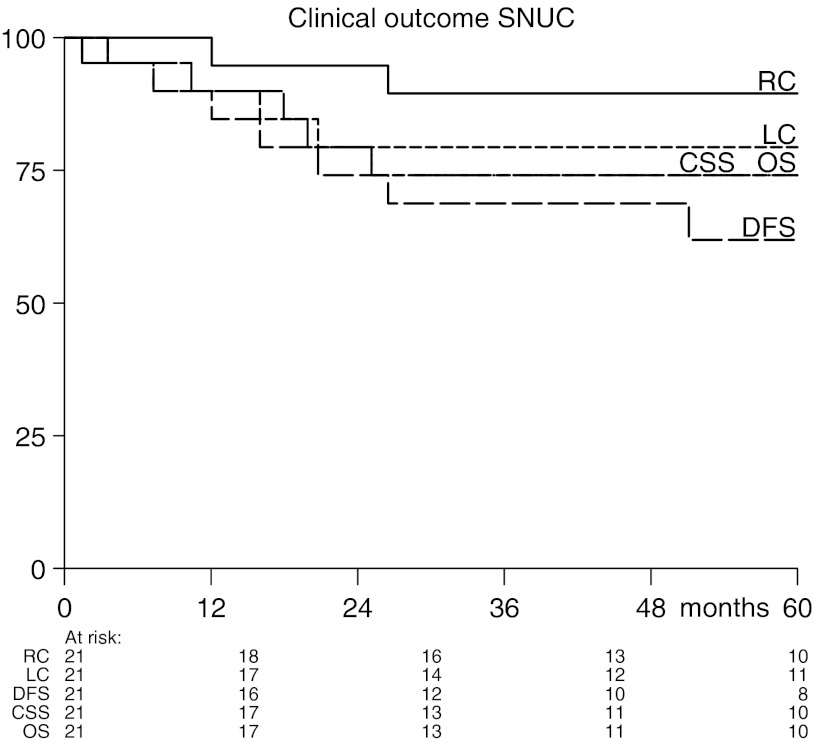
Kaplan–Meier curve of local control (*LC*), regional control (*RC*), cause-specific (*CSS*), disease-free (*DFS*) and overall survival (*OS*)

After a median follow-up of 54 months (range 4–163), the 5-year actuarial rates of LC, RC, DFS, CSS, and OS were 80, 90, 64, 74, and 74 % respectively (Fig. 1).

Twelve events were reported: eight in-field local (LF), two regional (RF), and two distant failures, resulting in absolute LC, RC, distant metastasis-free survival (DMFS), and DFS rates of 62, 91, 91, and 48 %, respectively. The median time from treatment completion to any progression was 16 months (range 4–144).

Two of LFs were successfully salvaged with surgery and postoperative stereotactic radiotherapy. One of them underwent orbital exenteration as a part of the salvage surgery. In other patients with LF (*n* = 6), local treatment was not possible and they died eventually because of local progression. The median time from the treatment failure to death was 4 months (range 1–13).

Two patients had node-positive disease; one patient underwent neck dissection (ND) at the same session of the craniofacial resection and the second patient received 70 Gy radiotherapy to the primary tumor and the involved neck. Of the 19 node-negative patients, 8 patients (42 %) received elective nodal irradiation (ENI) (46–50 Gy) to the ipsilateral neck (level I–III). Of the entire group, only two patients developed RF and none of them received ENI; both were successfully salvaged with ND and PORT (66 Gy).

Of the whole group, six patients (29 %) died; in all of them, the cause of death was cancer related: in five patients because of local progression and in one patient because of both LF and DM.

On univariate analysis, high T-stage, the use of two- instead of three-modality treatment approach, the presence of dural or orbital invasion, and the omission of surgical treatment were significantly correlated with poor LC. However, on multivariate analysis only the first two parameters were still independently correlated with poor LC (Table 2).

**Table 2:** Exact logistic regression analysis: correlation between different parameters and local failure

	UVA (*p* value)	MVA (OR and *p* value)
T-stage (T4 vs. T3)	**0.02**	**36 (0.002)**
N-stage (N+ vs. N0)	0.99	
Tumor site (ethmoid vs. maxillary)	0.68	
Dural or intracranial extension (yes vs. no)	**0.005**	NS
Surgery (no vs. yes)	**0.02**	NS
Treatment modalities (two vs. three)	**0.004**	**55 (0.0003)**
RT technique (2D and 3DCRT vs. IMRT)	0.17	
RT dose (≤60 Gy vs. >60 Gy)	0.76	

Significant *p* values are indicated in bold

*NS* nonsignificant *p* value; *LF* local failure; *UVA* univariate analysis; *MVA* multivariate analysis; *OR* odds ratio; *RT* radiotherapy; *2D* two-dimensional; *3DCRT* three-dimensional conformal radiotherapy; *IMRT* intensity-modulated radiotherapy

### Late Toxicity

The overall 5-year cumulative incidence of grade ≥2 late toxicity was 30 %. Ten serious complications were reported in six patients, 50 % of these complications were ocular including unilateral blindness (*n* = 2), lacrimal duct stenosis (*n* = 2), and ectropion (*n* = 1). Non-ocular complications were dysphagia (*n* = 2), xerostomia (*n* = 1), deafness (*n* = 1), and maxillary osteoradionecrosis (*n* = 1). The incidence of late toxicity was reduced over time with the introduction of highly conformal radiotherapy techniques as IMRT. The incidence of serious complications in patients treated with IMRT versus 2D and 3DCRT were 14 and 57 %, respectively. None of the 14 patients treated with IMRT developed treatment-related blindness, while two of seven patients (29 %) treated by means of 2D or 3DCRT developed unilateral blindness.

Of the entire group, six patients had permanent visual problems because of tumor and/or treatment-related factors: three patients underwent orbital exenteration during the primary or salvage surgery because of LF, one patient was inoperable and the eye globe was not functional any more, and two patients developed treatment-related blindness resulting in an ultimate organ preservation of 71 %. Organ preservation was significantly improved over time in patients treated by IMRT, compared to 2D and 3DCRT (86 and 14 %, respectively, *p* = 0.006), since no patient treated with IMRT developed treatment-related blindness, compared to two patients treated with 2D/3DCRT.

## Discussion

Since SNUC was first introduced in 1986, only seven studies reporting on the outcome of ≥10 patients have been published from different institutions in the USA and Australia. Gallo et al. [10] published in 1993 an immunohistochemical study on 13 patients with SNUC and reported briefly on the outcome of these patients treated at the University of Florence between 1970 and 1993, mainly with radiotherapy. Only one patient was treated surgically and four with non-platinum-based chemotherapy. The present study is one of the largest and is the first European study to date reporting on outcome and toxicity of patients treated at the Erasmus MC-Daniel den Hoed Cancer Center in the era of modern surgical and radiation techniques and platinum-based chemotherapy regimens.

Table 3 illustrates patients’ demographics and outcomes of eight studies where patients with SNUC were treated with combined-modality treatment. Our review included only studies where at least ten patients were treated because smaller series cannot provide the means to draw solid conclusions. Comparison between these studies is complicated by major differences with respect to patients’ demographics, the used staging system, end points and therapeutic strategies. Patients’ characteristics and outcomes of the current study have more similarities with the reported data from the University of Florida [6] and the M.D. Anderson Cancer Center [5]. Small differences were observed, for instance in the length of the follow-up time, type of chemotherapy used, and the percentages of patients having T4 tumor, who received chemotherapy and were irradiated by means of IMRT. As shown in Table 3, the outcomes in the current study compares favorably with those reported in the other five studies. Possible explanations are [1] that higher radiation dose and highly conformal radiation techniques were mostly applied in our study. Dose–response relationship might exist in case of SNUC [11]. In the present study, patients who received >60 Gy showed better LC rates, compared to a dose of ≤60 Gy (75 vs. 44 %), albeit statistically not significant. The same is true for patients treated with IMRT, compared to 2D or 3DCRT (77 vs. 38 %). In patients treated with 2D or 3DCRT, underdosage of the target volume should often be tolerated to prevent late toxicity. Dirix et al. [12] showed that IMRT, compared to 3DCRT, improved DFS (*p* = 0.02) and LC (*p* = 0.06) and decreased acute and late toxicity [2]. Relatively more T4 tumors and (when reported) more tumors with orbital and/or intracranial invasion were treated in other studies [3]. In our study, more patients received induction chemotherapy with down-staging and subsequently resection and PORT. In most of the reviewed studies, the benefit of implementation of aggressive surgery as part of the treatment modality was clearly demonstrated [2–8]. In our study, the patients who underwent surgical resection had significantly better LC than those in whom it was omitted (85 vs. 25 %, *p* = 0.005). Adding induction chemotherapy to achieve down-staging before surgery should be considered, when resectability upfront is questionable. Carful selection of patients who are more likely to benefit from an attempt at such an organ preservation approach is of paramount importance. The benefit of induction chemotherapy to reduce the incidence of DM was suggested by Rischin et al. [8], since none of the seven patients with SNUC treated with induction followed by concurrent chemotherapy developed DM (follow-up 8-62 months), whereas both patients treated with surgery and PORT developed DM at 8 and 20 months, respectively.

**Table 3:** Review of literature on treatment outcomes in SNUC

	DDHCC	UCSF [2]	UV [3]	UMAA [4]	MDACC [5]	UF [6]^a^	UC [7]	PMCCC [8]
No. of patients	21	21	20	19	16	15	14	10
Years of inclusion	1996–2010	1990–2004	1986–2000	1995–2008	1982–2002	1992–2005	1970–1999	1990–2002
Median age (years)	52	47	58	51	48	57	54	49
Median FU time (months)	54	58	80	21	81	30		
Median RT dose (Gy)	62.5	57	55	60		64.8	61	54
AJCC T4 (%)	71	81	73	84	69	100	63	90
Node positive (%)	10	10	13	21	0	13		30
Surgery (%)	62	90	55	53	63	66	64	20
Radiotherapy (%)	100	100	95	100	100	93	86	100
Chemotherapy (%)	76	62	80	84		47	43	70
2 year LC (%)	80	60		83			43	50
5 year LC (%)	80	56			79	78		
2 year RC (%)	94			50				50
5 year RC (%)	90	90			84	80		
2 year DMFS (%)								80
5 year DMFS (%)	90	64		35	75	82		
2 year OS (%)	74		47	61			45	64
5 year OS (%)	74	43	20	22	63	67		
5 year CSS (%)	74					77		

*DDHCC* Daniel den Hoed Cancer Center, Rotterdam; *UCSF* University of California, San Franscico; *UV* University of Virginia; *UMAA* University of Michigan, Ann Arbor; *MDACC* M D Anderson Cancer Center, Houston; *UF* University of Florida; *UC* University of Cincinnati, *PMCCC* PeterMac Callum Cancer Centre, Melbourne; *FU* follow-up; *AJCC* American Joint Committee on Cancer; *LC* local control; *RC* regional control; *DMFS* distant metastasis-free survival; *DFS* disease-free survival; *CSS* cause-specific survival; *OS* overall survival

^a^Three-year outcomes were reported

In the reviewed literature, the incidence of node-positive disease at presentation varied from 10 to 30 % (mean 13 %). In our study, 42 % of high-risk node-negative patients received ENI. None of them developed RF. Two RFs were reported in patients who did not receive ENI, resulting in regional control rates of 100 and 82 % for patients who did or did not receive ENI, respectively. Our results are comparable with those reported by the University of Florida (100 and 66 %) [6] and the University of California (94 and 75 %) [2]. However, Rischin et al. [8] reported a 50 % nodal recurrence rate. In that study, ENI was, to our knowledge, not applied. The question whether ENI should be given to all patients with SNUC is difficult to answer because in most of these studies ENI is recommended on a case-by-case basis. However, the results of the small studies (where ENI was applied) are encouraging with respect to regional control. Therefore, we would advocate ENI in patients with locally advanced SNUC. According to our local treatment protocol, ENI was advocated in T4 high-risk node-negative patients with involvement of the skin of the cheek, infratemporal fossa, or pterygoid or cribriform plates.

The good LC seen in our study was associated with a high rate of grade ≥2 late toxicity (30 %). However, the incidence of serious late toxicity was reduced over time with the implementation of IMRT compared to 2D and 3DCRT, albeit statistically not significant (14 vs. 57 %; respectively *p* = 0.2). Furthermore, the incidence of permanent visual impairment was significantly reduced in patients treated by IMRT compared to 2D and 3DCRT with an ultimate organ preservation rate of 86 and 14 %, respectively (*p* = 0.006). The improved therapeutic ratio achieved by the use of IMRT would allow us to escalate the dose of radiotherapy locally to further improve LC rates, since local recurrence is the most significant problem in SNUC.

### How to make progress?

Local recurrence and DM remain significant problems in patients with SNUC. Treatment strategies to improve outcomes should therefore focus on improving local and distant disease control. In a recent review of Robbins and colleagues [13], recommendations for the future direction of therapeutic investigations are outlined. According to that review, further progress in the treatment of SNUC could be achieved through the development of endoscopic surgery, high-precision high-dose radiotherapy, and further intensification of chemotherapeutic schedules. The slightly improved LC rates, the reduction in the late toxicity and permanent visual impairment, and subsequent improvement in the therapeutic ratio seen by the implementation of IMRT should allow dose escalation of radiotherapy to further improve LC, since dose–response relationship might exist in case of SNUC [11]. To further reduce the risk of late (especially ocular) toxicity, hyperfractionated scheme of radiotherapy, as applied to two-thirds of patients treated at the University of Florida [6], could be offered to patients treated with radiotherapy without chemotherapy, since acceleration of radiotherapy is probably not beneficial in concomitant chemoradiotherapy schedules [14]. Highly conformal new radiation techniques as proton therapy might offer new perspectives in the radiation treatment of SNUC. Mock et al. [15] showed that protons could achieve 60 % dose reduction in organs at risk, compared to IMRT while keeping similar or better target coverage. In patients were the respectability of the SUNC is doubtful, an attempt of down-staging by induction chemotherapy might be considered to subsequently improve LC, DFS, and OS. Carful selection of patients who were more likely to benefit from an attempt of down-staging before surgical resection is, therefore, of paramount importance. The randomized controlled study of Hitt et al. [16] showed that in patients with unresectable locally advanced head and neck squamous cell carcinoma, induction chemotherapy with paclitaxel, cisplatin, and fluorouracil resulted in an overall response rate of 80 %. Because limited evidence exists for taxanes in sinonasal malignancies, the optimal regimen for induction chemotherapy needs, therefore, to be explored in prospective studies. Other possible advantages of induction chemotherapy in these patients are the reduction of DM rate [17], symptomatic relief in patients in need of immediate therapy, and a logistical advantage to avoid unnecessary delay in starting radiotherapy because of the waiting time.

The limitations of the current study are well recognized by the authors. Inherent to the rarity of the disease, the number of patients treated is small and it is extremely difficult to conduct randomized or even large prospective studies to investigate different unclear issues with respect to the optimal treatment of SNUC. Although the conclusions drawn are of low level of evidence, the results of the present study will, in our opinion, strengthen the small bulk of evidence available in the literature about different issues of the management of these patients. The late toxicity was retrospectively scored using chart review only. Accurate assessment of less severe complications from the medical records is not really reliable because of the subjective nature of these end points. Therefore, it is likely that not all mild late toxicities were captured.

## Conclusions

In the current study, combined-modality treatment with three, or at least two, modalities for patients with SNUC resulted in good LC, but with high overall rate of late toxicity. However, the incidence of late toxicity and visual impairment were decreased over time by the introduction of IMRT. The reduction in late toxicity was also associated with slight improvement in LC rates, albeit statistically nonsignificant. The improved therapeutic ratio achieved by IMRT would allow dose escalation of radiotherapy and subsequently improve LC as the most significant problem in patients with SNUC. Therefore, highly conformal radiotherapy techniques as IMRT should be the standard of care in patients with SNUC.

Although all of the reported series in our review are small, some important conclusions can be drawn from the review of the literature. First, gross tumor resection should be considered in all patients, as better outcome has been noted with the implementation of aggressive surgery as part of the treatment modality. In case of doubt about the feasibility of primary surgical resection, induction chemotherapy would be offered to patients with good performance and where an attempt of organ preservation is reasonable. Second, a treatment schedule comprising three, or at least two, treatment modalities should be offered to all patients. The sequence of these modalities would be dictated by disease extent, performance state, and the available treatment resources. Furthermore, ENI would be advocated in all patients with locally advanced disease, as the results of the small studies where ENI was applied are encouraging with respect to regional disease control.
